# Neuromuscular Activity Determines, at Least in Part, the Motoneuron, Nerve and Muscle Properties Under Normal Conditions and After Nerve Injury

**DOI:** 10.3390/ijms26146891

**Published:** 2025-07-17

**Authors:** Tessa Gordon

**Affiliations:** Department of Surgery, Division of Plastic Reconstructive Surgery, University of Toronto, Toronto, ON M5G 1X8, Canada; tessat.gordon@gmail.com; Tel.: +1-647-678-1314

**Keywords:** fast- and slow-twitch muscles, neuromuscular activity, cross-reinnervation, muscle fiber types, gene expression, motoneuron properties, muscle properties, electrical stimulation

## Abstract

Whether *pattern* or *amount* of daily activity determines neuromuscular properties is the focus of this review. The fast-to-slow conversion of many properties of fast-twitch muscles, by stimulating their nerves electrically with the continuous low-frequency pattern typical of slow motoneurons, argued that muscle properties are determined by their *pattern* of activity. However, the composition of the motor units (MUs) in almost all muscles is heterogeneous, with the MUs grouped into slow, fast-fatigue-resistant and fast-fatigable types that match corresponding histochemical fiber types. Nonetheless, their contractile forces lie on a continuum, with MUs recruited into activity in order of their size. This ‘size principle’ of MU organization and function applies in normally innervated and reinnervated muscles and, importantly, begs the question of whether it is the *amount* rather than the *pattern* of the MU activation that determines their properties. Experimental evidence that uniform daily *amounts of* ~<0.5, 5%, and 50% ES, converted motoneuron, nerve, and muscle properties to one physiological and histochemical type, argued in favor of the *amount* of activity determining MU properties. Yet, that the properties were not confined to the expected narrow range argued that factors other than the *pattern* and/or *amount* of neuromuscular activity must be considered. These include the progressive increase in the synaptic inputs onto motoneurons. The range of the effects of endurance and intermittent exercise programs on healthy subjects and those suffering nerve injuries and disease is also consistent with the argument that factors other than *pattern* or *amount* of neuromuscular activity should be investigated.

## 1. Introduction

Mammalian fast- and slow-twitch skeletal muscles were first identified as red and white in the 1800s on the basis of their color [1,2] and fatiguability [3]. The high endurance of the slow-twitch muscles for repetitive muscle contractions [4] is associated with their high resting blood flow and oxidative/glycolytic ratios [5,6]. There are, however, few (if any) homogenous muscles with regard to the composition of their muscle fibers [7]. The majority of the fast-twitch muscles have a separate deep compartment, which contains slow oxidative (SO; type I) muscle fibers that are intermingled with fast-oxidative-glycolytic (FOG; type IIA) fibers, and a superficial and larger compartment in which the fast fibers of both FOG and fast-glycolytic (FG, type IIB) types reside [8,9,10,11,12]. The slow-twitch muscles, in contrast, are either homogenous, with slow-twitch soleus (SOL) muscles in cat hindlimbs and human legs having only SO fibers, or the muscles include a small number of FOG fibers [7]. These slow-twitch muscles are located deep within limbs and close to bone [8,10].

In the early 20th century, Sherrington [13] coined the term motor unit (MU) for the motoneuron and the muscle fibers it supplies. He referred to the MU as the final common pathway of the nervous system because all the processing in the central nervous system results in activation of the MUs and, in turn, movement. The MU includes the motoneuron (cell body, dendrites and axon) and the muscle unit (MusU), the muscle fibers it supplies. The MusUs were first divided into slow (S) and fast (F) on the basis of the twitch contractile speed [14]. Burke and colleagues then divided the F units in to fast-fatigue resistant (FR) and fast-fatigable (FF) MusUs in the cat medial gastrocnemius (MG) muscle on the basis of their fatigability and distinguished S from F MusUs by the absence and presence of ‘sag’ of their unfused tetanic contractions in response to the stimulus train of 25 pulses/s (second) rather than on their twitch contraction times [14,15,16]. The last MusU that was isolated and identified as S, FR or FF was stimulated repetitively, as described by Edstrom and Kugelberg [16], to deplete the MusU fibers for subsequent histochemical analysis and classification as SO, FOG or FG [17]. The excellent correspondence of the physiological and histochemical criteria in the isolated MusUs formed the basis of the Burke MusU classification that remains widely used in many studies [18]. The distinction of the muscle fiber types on the basis of their myosin ATPase is due to differences in myosin heavy chains, each of which is coded by a separate gene [18]. There are several, however, as there are of the myosin light chains [19]. The biochemical analysis of enzyme activities in single muscle fibers revealed a continuum of enzyme activities, but the MusU fibers are homogeneous with relatively little variance between them, as compared to the variance between muscle fibers of the same histochemical type 1 [20,21].

The *pattern of neuromuscular activity* was advocated by Vrbová and Pette as responsible for the plasticity that fast- and slow-twitch muscles display in their capacity to alter their properties in response to imposed activity by electrical stimulation [20,22,23,24,25]. On the other hand, the experiments of Kernell and colleagues [26,27,28,29] and Gordon and colleagues [30,31,32,33,34,35] provided equally compelling evidence that the *amount of neuromuscular activity* determines their properties, including those of the motoneurons and their motor nerves. This review concerns evidence for and against these two competing explanations for the differences in the neuromuscular properties. In addition, several other factors, including the intrinsic regulation of slow and fast muscle fiber phenotypes and different mechanical conditions, are imposed by their locations deep and more superficially, respectively [36,37,38,39].

## 2. Pattern of Neuromuscular Activity

### 2.1. Muscle Cross-Reinnervation

**Figure 1:**
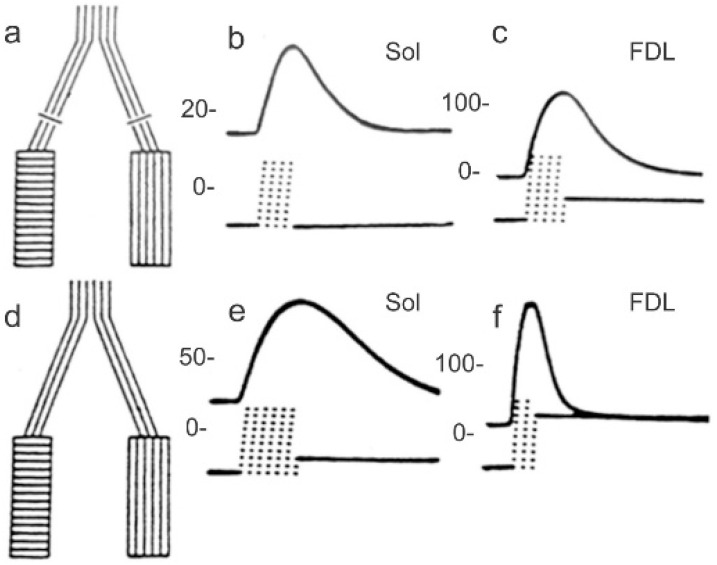
Cross-reinnervation of slow- and fast-muscles increases and decreases their twitch contraction times, respectively. (**a**) Figurative representation of the surgical cross-suture of the nerves to slow and fast muscles. Twitch contractions of cross-reinnervated (**b**) slow-twitch soleus (SOL) and (**c**) fast-twitch flexor digitorum longus (FDL) muscles. (**d**) Figurative representation of the normal innervation of fast and slow muscles. Twitch contractions of normally innervated (**e**) SOL and (**f**) FDL muscles. The scales of contractile force are in grams, and each dot below the twitch contractions represents 1 ms. Adapted with permission from [40]. Copyright 1960 The Physiological Society.

A seminal paper published by Buller, Eccles and Eccles in 1960 suggested that the muscle phenotype is determined by the motoneuron [40]. They used the experimental paradigm of cross-reinnervation of fast- and slow-twitch muscles in the cat hindlimb to examine whether and how the nerve determines the properties of the muscle that it supplies. The slow-twitch SOL muscle nerve was cut and sutured to the distal stump of the cut ‘fast’ nerve to one of several fast-twitch muscles in kittens and adult cats. Recordings of the isometric force of the cross-reinnervated muscles demonstrated that the apparent fast-to-slow conversion of the fast-twitch muscles, the time-to-peak force and the relaxation time of the twitch contraction slowed, and a slow-to-fast conversion of the slow-twitch SOL muscle. Yet, as illustrated in Figure 1, the conversions were incomplete with neither cross-reinnervated muscles, displaying the contractile speeds of the unoperated fast- and slow-twitch muscles [40].

### 2.2. Electrical Stimulation

**Figure 2:**
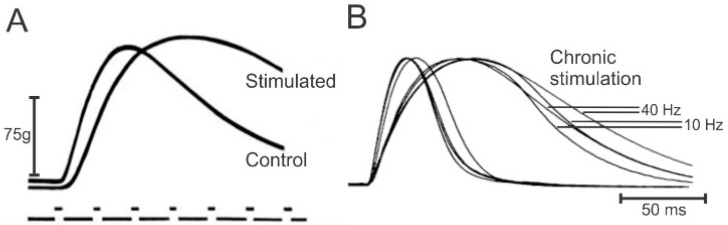
Fast–slow conversions of rabbit and cat fast-twitch muscles by (**A**) the *pattern* of low-frequency (20 Hz) continuous activity of slow motoneurons and (**B**) the *amount* of daily activity, regardless of the frequency of the activity. The muscle twitch contractions are shown for (**A**) control and stimulated tibialis anterior muscles and (**B**) control and stimulated PerL muscles. (The markers below the twitch contractions in (**B**) represent 10 msec intervals). Adapted with permission from [26,47], respectively.

Buller et al. [40] attributed the muscle speed conversions to “trophic” effects of the cross-reinnervating nerves but, they provided an alternate explanation of Huxley that the different activation patterns of low steady and the short-lived high-frequency firing rates typical of slow- and fast-MusUs, respectively [4,41,42,43,44,45], were responsible for the conversions. In support of Huxley’s explanation, (1) the SOL muscle became fast-contracting after eliminating the stretch reflex-induced muscle contractions by cutting the muscle tendon—tenotomy [46], and (2) the fast-contracting tibialis anterior (TA) muscle became slow-contracting in response to continuous low-frequency (10 Hz) electrical stimulation (ES) of the common peroneal (CP) nerve after all the natural neuromuscular activity of the hindlimb was eliminated by spinal cord transection at T11 and L1 levels (Figure 2A; [47]).

**Figure 3:**
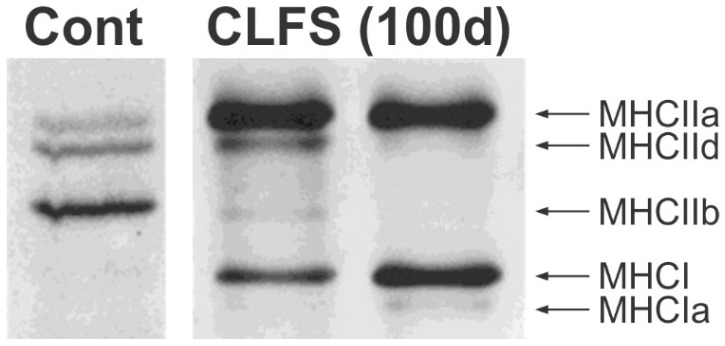
Chronic low-frequency electrical stimulation (CLFS) converts fast myosin heavy chains to slow. Electrophoretic separation of MHC isoforms from rat tibialis anterior (TA) muscles after 10 weeks of CLFS (2 h/day of 15 Hz at tolerable stimulation levels, for 2 s on and 4 s off). Adapted with permission from [19]. Copyright 2000 Wiley-Liss, Inc.

Extensive studies followed to demonstrate fast-to-slow histochemical, immunological and molecular conversions of fast-twitch muscles by continuous, low-frequency ES [23,24,25]. These included the fast-to-slow conversion of TA muscles with enhanced capillarization and blood flow, conversion from anaerobic to aerobic metabolism, fast-to-slow conversion of myosin isoforms, calcium regulatory proteins including sarcoplasmic reticulum Ca^2+^-ATPase, the α-subunit of the dihydropyridine receptor and calsequestrin, and the gene expression of troponin and tropomyosin in the sarcoplasmic reticulum [10,19,21,23,24,25,48,49]. For example, myosin isoforms transform from MHCIIb to MHCI and MHCIIa in rat TA (Figure 3; [19]) and from MHCIId/x to MHCI in human vastus lateralis muscle (VL) biopsies [50]. Within days of daily ES, mRNA levels [51,52,53] and activities of the enzymes of the citric acid cycle, fatty acid β-oxidation and the respiratory chain increase significantly [51]. The enzymes include the aerobic enzymes, succinic dehydrogenase and NADH, which increase concurrently with an increase in the number of mitochondria per muscle fibre Williams 1987 [52] and with the decline in anaerobic glycolytic enzyme activities, with the changes following an exponential time course [48]. Examples of increased oxidative enzyme activity with a concomitant decline in glycolytic enzyme activity were demonstrated in human VL muscle biopsies [50].

Lomo and colleagues, recognising the deficiency of research on the effects of a fast pattern of ES, used the denervated SOL muscle as the model in which to compare the effects of ES in the continuous slow and high frequency stimulation patterns and those of ES in an intermittent high frequency pattern [54,55]. The investigators independently varied the number and frequency of the electrical pulses that were imposed by the chronic stimulation. Their conclusions were that large numbers of stimulus pulses had a slowing effect on the denervated SOL muscle that was greatest at the low firing frequencies of 10–20 Hz [55,56], typical of the normal SOL MUs [56,57]. Consistent with this conclusion are the findings that SOL muscle contractions become faster when the ‘amount of activity’ is reduced by limb immobilization [58,59], spinal cord transection [60] and denervating antagonistic muscles [61,62].

## 3. Amount of Neuromuscular Activity

### 3.1. Kernell’s Experiments

**Figure 4:**
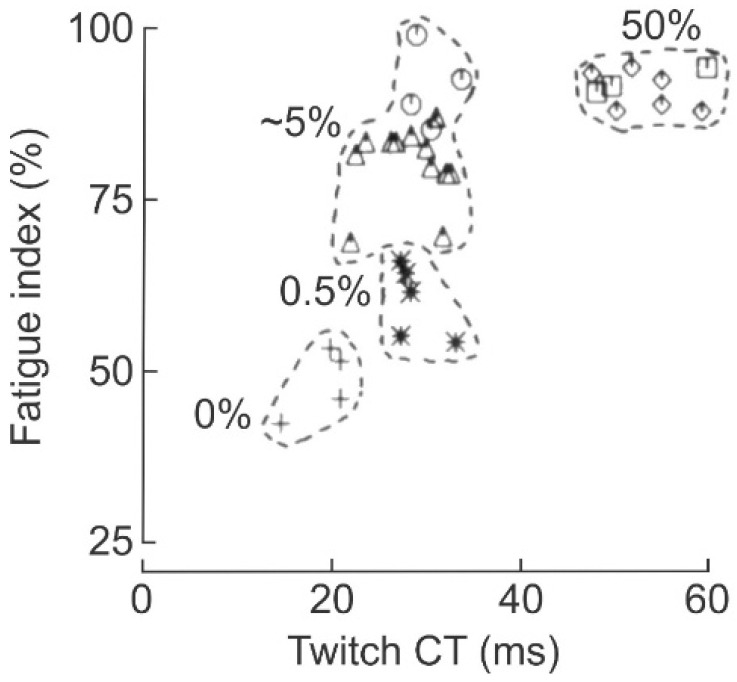
The contraction speed and fatigability of hindlimb muscle increase with the daily *amount of neuromuscular activity.* The fatigue index (FI) as a percent of maximum, the maximum being “no fatigue”, is plotted as a function of the twitch contraction time (CT). The data was obtained in different paralyzed cats in whom the daily *amount* of electrical stimulation (ES) of the peroneal longus (PerL) muscle was <0.05%, ~5% and 50% of each day for 4 to 8 weeks. The muscles stimulated for 4 weeks are represented by plus signs, stars, open circles and open squares and those stimulated for 8 weeks by open triangles and diamonds. The dashed lines surround the muscles that were subjected to 0%, 0.5%, ~5%, and 50% *amounts* of ES. Graph drawn from data from [28,29].

Comparisons of the effects of daily low (10 Hz) and high (40 Hz) frequencies of ES of the nerve to the paralyzed peroneus longus (PerL) muscle in cats demonstrated that the two frequencies were equally effective in fast-to-slow conversion of the muscle (Figure 2B; [26,28]). PerL muscles were paralyzed by hemisection of the spinal cord at L1 and deafferentation of the lumbosacral motoneurons (HSDA) in order to implement ES for a duration of 50% of each day [26,27,28,29]. Daily 50% ES in an on–off pattern that continued throughout each day (either at a continuous 5 Hz frequency or in 10, 20 or 40 Hz intra-burst rates on and off each for 1 s) resulted in their conversion to slow-contracting, fatigue-resistant muscles (Figure 4; [28,29]). The fast-to-slow muscle conversion was demonstrated by the increased twitch contraction time (CT) and fatigue index (FI) of the stimulated muscles where the FI is the ratio of tetanic forces developed at the start and the end of a two-minute period of repetitive 40 Hz stimulation of the CP nerve for 300 ms of every second. The slowing and reduced force of the stimulated PerL twitch contractions occurred at all the ES frequencies [26], and they were accompanied by the corresponding conversion of fast-to-slow fiber types and reduction in the size of their muscle fibers [27]. This demonstration of the role of the *amount* rather than *the pattern* of daily neuromuscular activity in controlling muscle properties was further clarified by their findings on the conversion of the properties to those typical of fast-contracting, fatigue-resistant muscles by reducing their daily activity to 5% of each day (Figure 4). When the daily amount of activity was reduced further to 0.5% and <0.5%, the PerL muscles were converted to the FG and FF phenotypes, respectively (Figure 4).

### 3.2. The Size Principle

Introduced by Henneman in the 1960s [63,64,65,66], the progressive activation (recruitment) of MUs during movement in order of size, with respect to nerve conduction velocity (CV) and MusU force, is consistent with the *amount* of activity determining muscle properties. This size-dependent MU recruitment was demonstrated in both cats [63,67,68,69] and human subjects [70,71].

**Figure 5:**
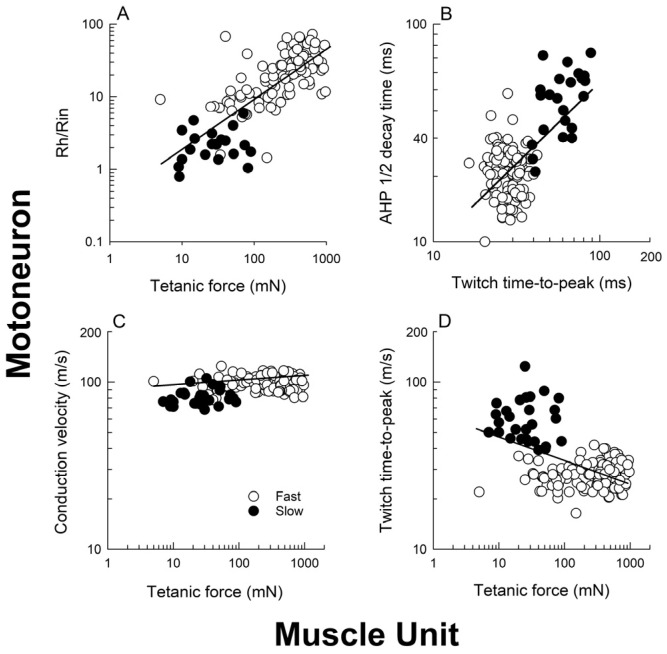
Size-dependent matching of the electrical and contractile properties of motoneurons and their muscle units, respectively. Correlations of (**A**) the ratio of rheobase current (Rh) to input resistance (Rin) of medial gastrocnemius (MG) motor units as a measure of their excitability, and their muscle unit (MusU) tetanic force; (**B**) half-decay time of the after-hyperpolarization (AHP) of the MN action potential and the time-to-peak force of the MusU twitch contractions (TTP); (**C**) conduction velocity of the motor nerves to the MG muscle and the tetanic force of the MG MusUs they supply. The inverse correlation of (**D**) the MusU twitch TTP and tetanic force. The correlation coefficients of all the regression lines were significantly different from zero (*p* < 0.05). The open and closed circles represent fast and slow MusUs defined by their presence and absence of ‘sag’ of their unfused tetanic contractions. Adapted from [72].

**Figure 6:**
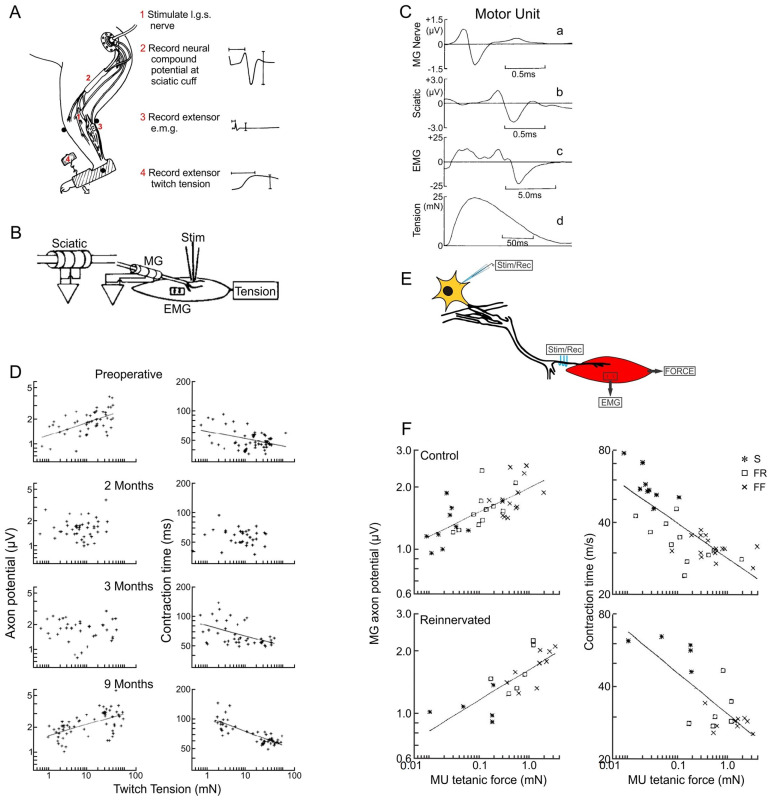
Normal and reinnervated muscles obey Henneman’s size principle. (**A**) Chronic recordings of sciatic nerve compound action potentials in response to stimulation of the medial gastrocnemius (MG) nerve, the electromyographic signal (EMG) from MG muscle and, by coupling the foot to a strain gauge, the MG muscle isometric contractile force. (**B**) Diagrammatic representation of the chronic recording of unitary MG nerve fiber action potentials on triphasic electrode arrays within silastic cuffs that surrounded the MG and sciatic nerves in response to electrical stimulation of one MG nerve fiber at the muscle’s motor point by a fine bipolar needle electrode. (**C**) The motor unit’s unitary action potentials on (a) MG and (b) sciatic nerves and (c) EMG on bipolar muscle electrodes and (d) muscle force that were evoked in an all-or-none manner by the intramuscular stimulation of one MG nerve. The unitary responses were identified by all-or-none responses. (**D**) The linear relationship of MG nerve action potentials and MU twitch forces and the inverse relationship between the muscle unit (MusU) contraction time and twitch isometric forces before and months after MG nerve transection and surgical repair. Individual recordings are shown as + symbols and the regression lines were drawn only when the slope of the lines was significantly different from zero at *p* < 0.01. (**E**) Diagrammatic representation of acute recording in uninjured control and experimental cats (1–2 years post-surgical MG nerve transection and repair) from MG nerve and muscle in response to stimulation of a single motor nerve in a teased ventral root filament. (**F**) MG axon potentials and MusU contraction times are plotted as a function of the contractile force of the MusU types of S, FR and FF, identified by different symbols. Regression lines were drawn in the graphs only when the slope of the lines was significantly different from zero at *p* < 0.01. Adapted from [74,75].

The CVs of the cat MG motor nerves, the electrophysiological measure of the size (and diameters) of nerves supplying S and F MusUs, increase from the S to the F as a function of their MusU contractile force in the MG muscle, but differences in the nerve CVs within the F group were not discernable (Figure 5C; [72]). Yet, in support of the findings of Henneman and colleagues [64,65,66], Jami and Petit [73] reported near-linear relationships between logarithmic values of tetanic tension of MusUs and the CV of their nerves in four cat hindlimb muscles, including SOL and TA. Chronic recordings of unitary action potentials on the MG nerve indicated that the peak-to-peak amplitudes of these potentials were a more reliable electrophysiological measure of nerve size (Figure 6A,B; [74,75]). The size of individual motor nerves was also measured by the peak-to-peak amplitudes of unitary action potentials that were evoked by the stimulation of single motor nerves via a bipolar needle electrode inserted into the muscle at or near the motor point (Figure 6C,E; [74]). The electrical parameters of rheobase current (Rh) and input resistance of motoneurons (Rin), obtained with intracellular stimulation and recording of the motoneurons with a glass microelectrode, are good measures of motoneuron size [76,77]. Rh is the electrical current that evokes a nerve fiber action potential and is, by definition, an index of motoneuronal excitability whose value increases as the size of the motoneuron increases. Rin decreases with the neuron’s surface area, such that Rin values decrease with size, being highest in the small motoneurons. It is the Rh/Rin ratio that provides the excellent electrophysiological measure of MN size (Figure 5A; [72]).

**Figure 7:**
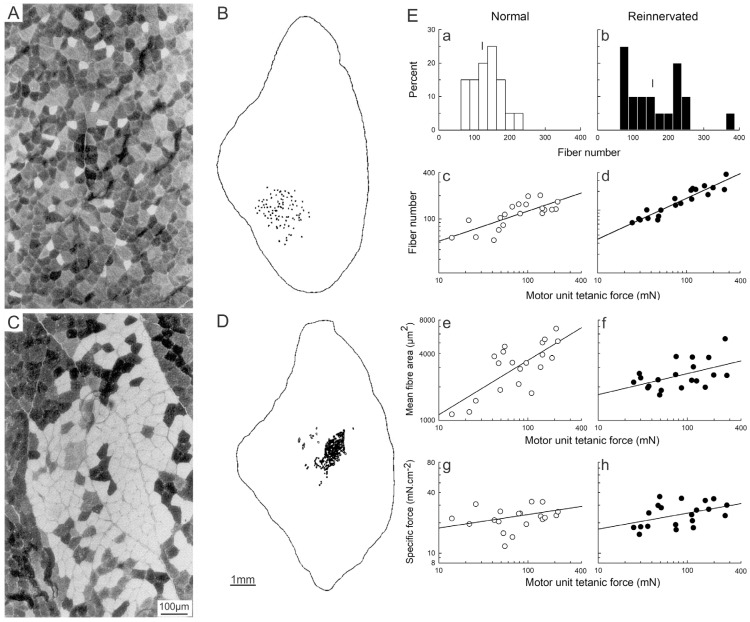
The number of muscle fibers supplied by each motoneuron, and to a lesser extent, their size, determine their contractile force. The white glycogen-depleted muscle fibers within a muscle unit (MusU) are the fibers innervated by one motoneuron in rat tibialis anterior muscle cross-sections of (**A**) normally innervated and (**C**) reinnervated muscles after common peroneal (CP) nerve transection and surgical repair. Camera lucida drawings of the total number and distribution of the MusU fibers of the (**B**) normally innervated and (**D**) reinnervated muscles. (**E**) Numbers of MusUs plotted as histograms and (**a**–**d**) as a function of motor unit (MU) force. Mean MusU fiber area (**e**,**f**) and MusU fiber specific force (**g**,**h**) are plotted as a function of MU force in normally innervated and reinnervated TA muscles. The bars (|) on the top of the histograms are the mean values of the percent distributions. The slopes of the regression lines were significantly different from zero (*p* < 0.05) with the exception of the lines drawn for the plot of fiber-specific forces as a function of MU force. Adapted from [78].

Henneman and colleagues [63,64] had assumed that MusU contractile force reflects their innervation ratio, namely the number of muscle fibers innervated by each MN, in addition to the cross-sectional area of the muscle fibers. This assumption was proven to be correct by experiments performed in Gordon’s laboratory [78], in which an identified MusU in rat TA muscles was classified as S, FR, FI or FF on the basis of both the CT of the twitch contraction and the fatigue index (FI) prior to its repetitive stimulation to deplete the MusU of glycogen. Using histochemistry to locate, type, count and measure the cross-sectional area of the glycogen-depleted MusU fibers, the authors confirmed the assumptions made by Henneman and colleagues [64,65] that MusU force indeed increases with the number of the MusU fibers and, to a lesser extent, with the total cross-sectional area of the fibers (Figure 7c,f; [78]).

In summary, the relationships between multiple measures of MN, nerve, MusU force and innervation ratio all provide strong evidence of Henneman’s size principle.

### 3.3. The “Speed” Match of Motoneurons and Their Muscles

**Figure 8:**
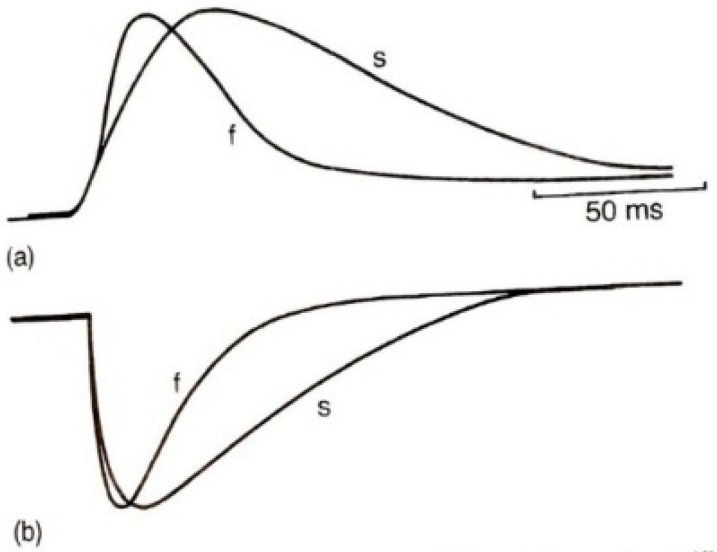
Matching of the time course of the twitch contraction of fast and slow muscle units in rats’ medial gastrocnemius muscle and the after-hyperpolarizations that follow the action potentials of the motoneurons that innervate them. (**a**) The isomeric twitch contractions of fast (f) and slow (s) muscle units and (**b**) the afterhyperpolarizations (AHP) that follow the action potentials of the motoneurons that innervate them. Their amplitudes have been normalized and are shown on a common time scale. Adapted from [85].

Grützner [2] recognised in 1884 that the slow-twitch muscle contractions fuse to a tetanic contraction at lower rates of nerve electrical stimulation than the twitch contractions of fast-twitch muscles. The fusion frequency of SOL muscle contractions was reported to be 30 pulses/s as compared to over 100 pulses/s for the contractions of fast-twitch gastrocnemius and flexor hallucis longus muscles in the cat [79,80]. Both the minimum and maximum rates at which motoneurons generate action potentials correlate with the duration of the twitch of the MusU fibers, that is, the discharge rates are low for the slow-contracting MusUs and progressively higher as the contraction times increase [45,81]. The minimum discharge rates that were recorded from motoneurons when they start to fire action potentials are typically those rates at which the MusU twitch contractions just begin to fuse to tetanic contractions [45]. Eccles and colleagues [82] made the classical observation that was confirmed later by others (e.g., [83,84]), namely that the duration of the after-hyperpolarization (AHP) of the action potentials generated by the motoneurons innervating slow muscles is longer than that of the motoneurons supplying fast-contracting muscles. These observations pre-empted the recognition of a ‘speed match’ between the duration of the motoneuron’s AHP and the duration of the MusU twitch contraction [41,84] (Figure 5C and Figure 8 [72,85]). The ‘match’ is continuous for rat MG MUs [85] but is less obvious for TA Mus, where the range of MusU contractile speeds is more limited [86]. The match is likely to be important for ensuring that the barely recruited motoneurons start to fire at the frequency that is optimally suited for the subsequent gradation of force. The S-shaped T-f relationship between muscle tension (T) and the frequency of stimulation (f) is steep and shifts to the right to higher frequencies for MusUs with faster contractile speeds (cf. Figure 5.2 in [18,81,83,85,86]).

**Figure 9:**
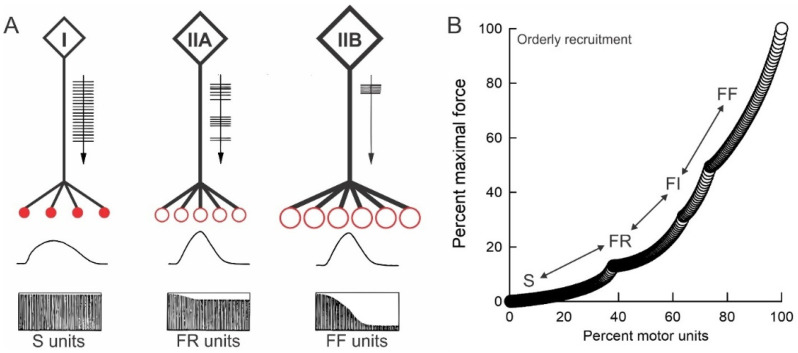
(**A**) Motor unit types and their orderly recruitment. Diagrammatic representation of type I (slow: S), type II (fast-resistant, FR) and type III (fast-fatigable, FF) motor units labelled on the basis of their physiological and histochemical properties. The pattern of firing of the units is shown and the increasing number of muscle fibers innervated by each motoneuron is represented. The muscle fibers of the type I and type II motor units are shown as filled and unfilled circles. The different sizes of the fibers are represented by the size of the circles. The slow and fast contractions of the S and the F units are shown and, below this, the tetanic contractions elicited at 40 Hz for 2 min, in the fatigue-resistant S and FR and the fatigable FF motor units. (**B**) Tetanic forces are plotted as a function of the numbers of the S, FR, FI and FF units in the cat medial gastrocnemius muscle, with both the forces and numbers as a percentage of the maximum force and numbers, respectively. The motor units are recruited in order of their size, with the muscle force increasing as a function of their number. See text for details of methods to delineate the different motor unit types according to the criteria of Burke et al. [16].

The low and continuous discharge rates of the ‘slow’ motoneurons contrast with the intermittent discharge of the ‘fast’ motoneurons (Figure 9A) as described in Section 2.2: [4,41,42,43,44,45]. Human SOL MusUs exhibit lower steady states of ~16 Hz compared to the higher maximal steady-state discharge rates of ~23 Hz of fast-twitch MG and LG muscles [87,88,89,90]. The low discharge rates allow postural firing for long periods without fatigue, while the higher, intermittent discharge rates of the large MusUs allow for the transient development of large forces in the muscles that fatigue more readily (Figure 9A; [16,67]). The fatigability of the forceful FF MusUs is well matched to the transient firing of their motoneurons due in part to their property of adaptation, namely the decline in their discharge rates during repetitive activity [18,67,91]. The rates decline with the slowing of the MusU contractions and a decline in their fusion frequencies [16]. The result is that the firing rates and the fusion frequencies remain well matched.

Threshold levels for MU recruitment during movement are different for different muscles. For example, they are lower in SOL as compared to MG and LG muscles in human subjects [92,93]. In addition, the control of the discharge rates of the motoneurons, namely, rate coding, contributes to the development of muscle force, with the relative contributions of MU recruitment and rate modulation varying in different muscles [44,94,95]. For example, rate coding accounts for a large component of force production in small foot muscles [87,96], in contrast to the gradual recruitment of MUs that occurs in the cat SOL muscle with almost no concurrent modulation of the MU firing rates [97,98]. While there are context-specific and muscle-dependent differences in the neuromechanical control of the triceps surae musculature [90], the orderly recruitment of MUs in order of their size is obeyed in all tasks (e.g., [99,100]).

### 3.4. The Size Principle After Nerve Injury

The size relationships between motoneurons, their nerves and the MusUs return in cat and rat hindlimb muscles after partial [101] and complete nerve injuries (Figure 6D,F; [74,75,102,103,104]). This was so, despite the regenerated nerves not reinnervating muscle fibers that they had supplied prior to the injuries. The relationships also returned in the hand muscles of patients with pressure or entrapment neuropathies and after microsurgical repair of transection injuries [105,106]. The size relationships returned even when the muscle reinnervation was functionally inappropriate, as demonstrated in reinnervated MG and TA muscles in cats after cross-suture of their nerves [103,107]. The relationships also returned after partial nerve injuries [101], where axons sprouted from remaining intact nerve to reinnervate adjacent muscle fibers after partial nerve injuries [8,107,108,109,110]. The size principle was restored in self-reinnervated cat triceps surae muscles [74] as shown by the significant regression lines drawn in Figure 6D. However, the chronic recordings of unitary nerve and MG muscle responses to the stimulation of single intramuscular motor nerves, using an inserted concentric needle electrode at or near the motor point (Figure 6B; [74]), revealed a delay in the return of the size relationships between the nerve action potential amplitude, isometric MusU twitch tension and MusU twitch contractile speed (Figure 6D; [74]). The early loss of the significant regression lines 3 months after the surgical nerve transection and repair reflects the random reinnervation of denervated muscle fibers by the regenerating nerves, as illustrated by the obviously changed muscle fiber distributions of glycogen-depleted MusUs in control and reinnervated rat muscles (Figure 7A–D; [78]). Initially, the reinnervated MusUs are heterogeneous in the muscle fiber-type composition as a result of the random reinnervation of denervated muscle fibers by the regenerating nerves. The delayed return of the size principle reflects the conversion of *all* the muscle fibers in the reinnervated MusUs to one type by their regenerated nerves [74,111,112,113,114].

### 3.5. Activity-Controlled Muscle and Motor Unit Properties

We expanded the Kernell studies of how the *amount* of neuromuscular activity affects PerL muscle and muscle fiber phenotypes [25,26,27,28,29] to investigations in cat and humans on the effects of (1) 0% daily neuromuscular activity on cat paralyzed hindlimb nerves [34], (2) 5% daily activity on paralyzed cat TA and MG muscles and MusUs [35], <0.5 to 5% daily activity on paralyzed TA muscles in spinal injured patients [115] and 5% daily activity on paralyzed MG muscle and MusU properties [35] and (3) 50% daily activity on paralyzed cat hindlimb muscle MG motoneurons [32] and their MusU properties [31].

#### 3.5.1. Reduction in Daily Neuromuscular Activity to 0%

**Figure 10:**
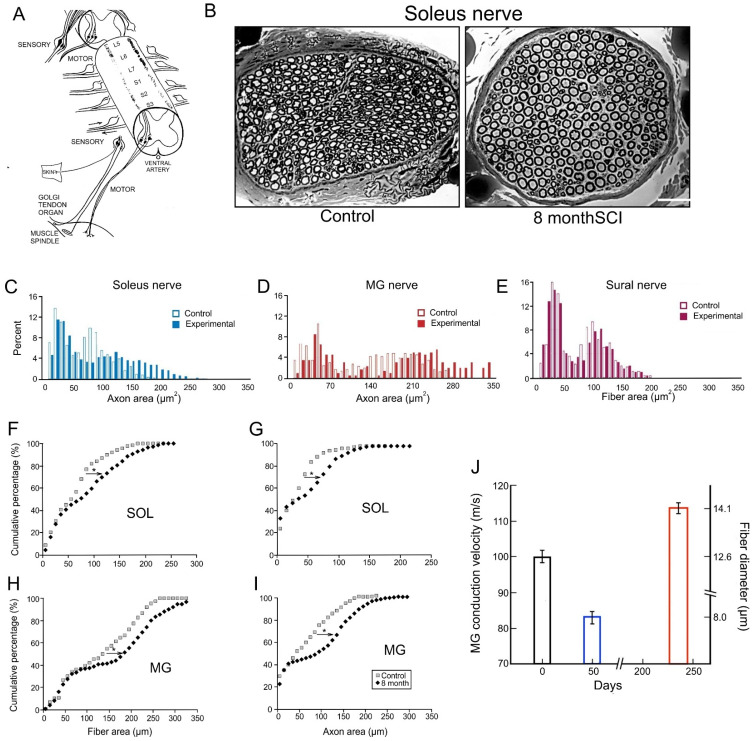
Elimination of motor nerve activity in cat hindlimb motoneurons by spinal cord isolation for 8 months increases the size of the nerve fibers. (**A**) Spinal cord isolation surgery (SCI). (**B**) Cross-sections of soleus (SOL) nerves 8 months after SCI and from the control nerve in the contralateral hindlimb. Histograms of axon areas of the nerves of (**C**) control and experimental soleus (SOL, after 8 months SCI) and (**D**) medial gastrocnemius (MG; 8 months after SCI). (**E**) Histogram of fiber areas of control and experimental sural nerves, 8 months after SCI. SOL (**F**) nerve fiber and (**G**) axon areas and MG (**H**) nerve fiber and (**I**) axon areas of control and experimental nerves, plotted as cumulative histograms. The rightward arrows with the asterisk (*) show the significant shift of the cumulative plots to the right to larger values (*p* < 0.01). (**J**) Histograms comparing the mean [±standard error (SE)] values of MG nerve fiber diameters and conduction velocities in cats experiencing normal neuromuscular activity, 50% daily electrical stimulation (ES) in a 50% duty cycle, (2.5 s on and 2.5 s off for 5 days/week) and little or no neuromuscular activity after SCI surgery (shown in black, blue and red, respectively). The scale bar in (**B**) is 50 µm. Adapted from [34].

Spinal cord isolation (SCI) surgery was performed to silence cat motoneurons and their nerves to hindlimb muscles, namely transection of the spinal cord above and below the motoneurons and the dorsal roots that enter the isolated cord, leaving the cell bodies in the dorsal root ganglia intact (Figure 10A; [34,116]). After 8 months, the axon and fiber size of the silenced MG and SOL nerve fibers, but not of the sensory sural nerves, increased significantly [34]. This significant shift was evident when axon and fiber areas were displayed as histograms or as cumulative histograms (Figure 10C–I; *p* > 0.01). The cumulative histograms were shifted to the right in parallel, excluding the fiber and axon areas of the smallest SOL and MG nerves that did not change, with these nerves being the afferent nerves from their muscle spindles and tendon organs (Figure 10F–I; [34]). These small SOL and MG nerves were not affected by their silencing in the same manner as the sensory SUR nerves were not affected (Figure 10E; [34]). The increased size of the motor fibers in silenced MG and SOL nerves support the hypothesis that low daily amounts of neural activity in the largest motoneurons and their motor nerves are responsible, at least in part, for their size, consistent with Henneman’s size principle [63,67].

#### 3.5.2. Low Daily Amounts of Neuromuscular Activity in Cat and Human Muscles

**Figure 11:**
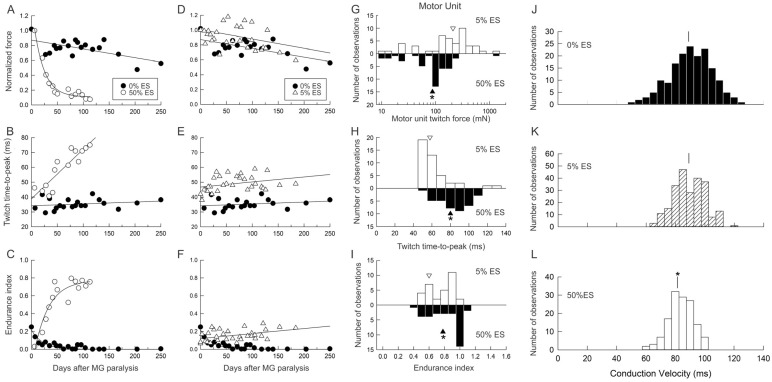
Daily nerve electrical stimulation delivered for 5% and 50% of each day elevates muscle and motor unit endurance while maintaining and reducing their conduction velocities and contractile forces, respectively. (**A**,**D**) Isometric tetanic force, normalized to the same day of daily electrical stimulation (ES); (**B**,**E**) the time-to-peak twitch force and (**C**,**F**) the endurance index of the cat medial gastrocnemius (MG) muscle as a function of the days after initiating (**A**–**C**) daily ES for 50% of each day (50% ES; ○) as compared to controls in which no daily ES was imposed (0% ES ●); (**D**–**F**) 5% ES (open triangle) of paralyzed MG muscle as compared to the controls in which no daily ES was imposed (0% ES ●). The paralysis was induced by hemisection at the T_12_–T_13_ level of the spinal cord and deafferentation by cutting the lumbosacral dorsal roots [35]. Histograms of motor unit (**G**) Tetanic force. (**H**) Time-to-peak twitch contraction and (**I**) endurance index for 5% ES and 50% ES. Mean values are denoted by inverted open triangles for daily 5% ES and by filled triangles for daily 50% ES on each of the histograms. Histograms of conduction velocities of MG nerves with (**J**) no daily ES (0% ES), (**K**) after daily 5% ES and (**L**) after daily 50% ES. Mean values are denoted by the vertical bars (|) on the three histograms with the significant difference in the mean values after 50% ES shown as an asterisk in (**L**) as compared to the mean values after no ES (0% ES) and 5% ES in (**J**) and (**K**), respectively. Adapted from [35].

**Figure 12:**
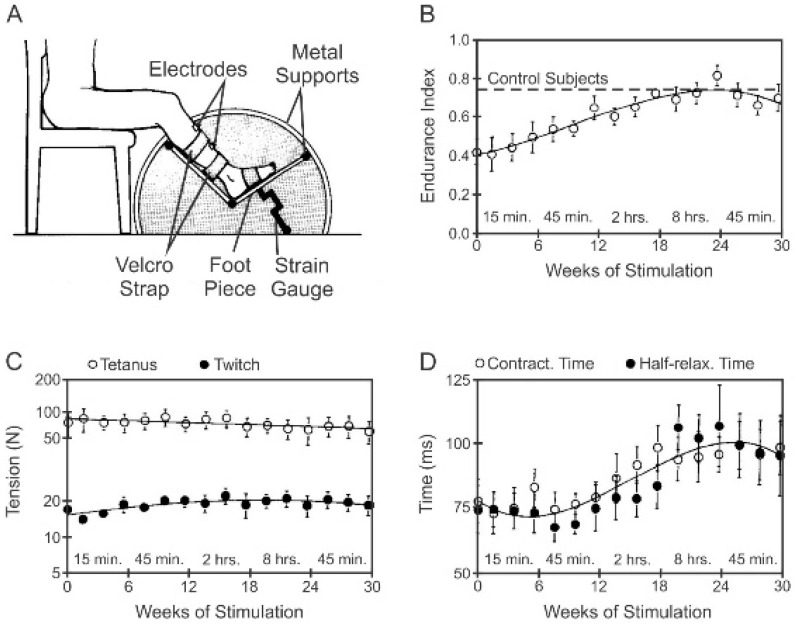
Muscle endurance of paralyzed tibialis anterior muscle increases in spinal injured subjects as the amount of daily 20 Hz electrical stimulation increases from 15 min to 2 h per day. (**A**) One leg was stabilized on a platform that was linked to a strain gauge for weekly recordings of isometric twitch and tetanic forces developed in response to stimulation at the motor point of the tibialis anterior muscle. The effect of progressive increase in the duration of daily ES on mean [±standard error (SE)] values of (**B**) endurance index, the ratio of force developed after and before 3.5 min of intermittent generation of tetanic contractions. (**C**) Twitch and tetanic isometric forces and (**D**) the time course of twitch contractions. Adapted from [115].

Paralysis of cats’ hindlimb muscles by the HSDA surgical paradigm of Kernell and colleagues [26,27,28,29] did not reduce the contractile force of the MG muscle recorded over time (Figure 11A,D; [33,35]). Similarly, the contractile force developed by stabilized TA muscles of spinal injured patients was not affected by their paralysis (Figure 12C; [115]). There was also little change in the contractile speed of twitch contractions in either paralyzed cat MG or TA muscles [35], as shown for the MG muscle in Figure 11B,E and Figure 12D [35]. In contrast, the endurance of the paralyzed muscles in the cat declined to very low levels (Figure 11C and Figure 12C; [33,35]). This fatiguability of paralyzed muscle was also reported in paralyzed human SOL [117,118] and quadriceps [119,120] muscles with accompanying type I-to-II-fiber-type conversion [113,121,122,123,124]. The ‘normal’ muscle forces recorded from the paralyzed cat MG and human TA muscles may be attributed to the remaining ~25% of neuromuscular activity [33] and the length of the muscles [125,126]. Muscle atrophy does occur in several paralyzed human muscles [125,126,127,128], developing with time after spinal cord injury [125,127]. The atrophy is severe in thigh muscles [122,126] as compared to the lesser atrophy of lower limb muscles such as the TA [113].

**Figure 13:**
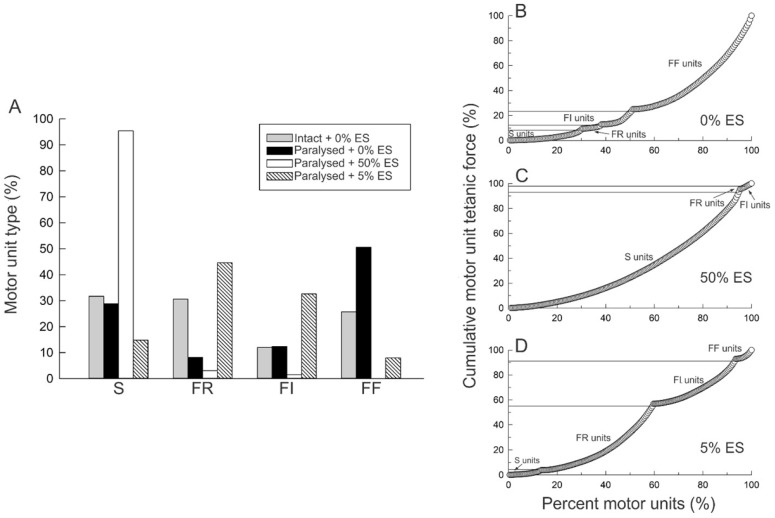
Increasing daily amounts of neuromuscular activity from 0. to 50% by 20 Hz electrical stimulation promotes a progressive slow-to-fast conversion of motor unit properties. (**A**) Percentages of motor unit (MU) types in the medial gastrocnemius (MG) muscle of cats paralyzed by hemisection and deafferentation (HSDA) either with no electrical stimulation [ES (paralyzed + 0% ES; *n* = 194], and the tibial nerve electrically stimulated daily with 5% ES: paralyzed + 5% ES; *n* = 224), or with 50% ES (paralyzed + 50% ES; *n* = 194). These percentages are compared directly with the percentages of MU types in intact cats (intact + 0% ES; *n* = 183). (**B**) Cumulative MU tetanic force as a percentage of maximum, plotted as a function of the percent of MU types in MG muscles, after 0% ES, (**C**) daily 50% ES and (**D**) 5% ES for 186, 138 and 198 days, respectively. Adapted from [35]. See text for details.

The contractile forces of both human and cat paralyzed hindlimb muscles and the time course of their contractions were not changed significantly by the daily *amounts* of 5% activity or less that were provided by electrical stimulation of their nerves at 20 Hz (ES) (Figure 13A–D; [35,115]). The ES was delivered to the patients’ TA muscles via external electrodes that adhered to the motor point [115] and, in the cats, via implanted electrodes around the CP and/pr MG nerves that were externalized for daily ES and regular monitoring of TA and MG muscle contractions [31,35]. ES was performed at home by the patients for progressively longer periods of 15 min (min), 45 min, 2 h, 8 h and, finally, 45 min each, for a 6-week period, i.e., 0.01%, 2%, 4%, 17% and 2% daily *amounts* of neuromuscular activity [115]. The ES regimens of daily *amounts* of 5% and 50% activities in the cats were 2.5 s (second) on and 2.5 s off for 3 h/day for 5 days/week [35] and 5 s on and 5 s off for 24 h/day for 5 days/week [31], respectively.

Daily 5% ES, both in human and cat muscles, having the dramatic effect of reversing the high fatigability of the muscle contractions (Figure 11F and Figure 12B), demonstrated that the *amount* of 4–5% daily activity is effective in promoting increased muscle endurance without compromising contractile force [33,35,115]. The MusU types shifted from FF to FR in the cat MG muscle without changing the proportions of the S-type MusUs or of SO muscle fibers (Figure 13A; [115]. The percentage of the FOG muscle fibers increased as the percentage of FF fibers declined (Figure 13A), consistent with the findings of Kernell and colleagues that 5% daily activity converts paralyzed PerL muscles to the FR muscle type [28,39]. The FR and FI MusUs almost entirely replaced the FF MusUs after 5% daily activity, with the FR MusU proportions doubling from ~25% of the MusUs to ~55% (Figure 13B,D). This conversion of FF to FR and FI MusUs by the 5% ES paradigm allows for the recruitment of ~55% of the MusUs during movement without muscle fatigue (Figure 13D).

#### 3.5.3. High Daily Amounts of Neuromuscular Activity

**Figure 14:**
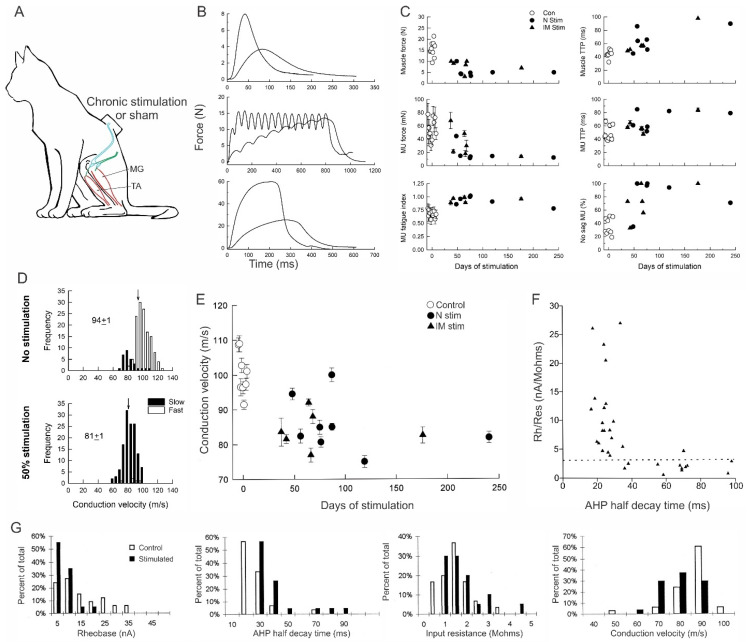
Motoneuron and muscle contractile properties are converted in parallel to a slow-type phenotype daily 50% daily neuromuscular activity. (**A**) Diagrammatic representation of the chronic electrical stimulation of the nerve to the medial gastrocnemius (MG) muscle for 50% of each day in cats. (**B**) Muscle isometric twitch and tetanic forces 73 days after initiation of chronic 50% ES. The ‘sag’ of unfused tetani elicited by pulse intervals of 1.25× time to peak values before initiating daily 50% ES changed to the ‘no sag’ characteristic of slow-twitch muscles (**C**) Muscle and motor unit (MU) twitch force, the time-to-peak twitch force (TTP), MU fatigue index and presence or absence of ‘sag’ are plotted as a function of the days of ES. (**D**) Histograms of MG nerve conduction velocities with mean values denoted by a arrow. (**E**) MG nerve conduction velocity plotted as a function of days of stimulation. Muscle and motor unit (MU) tetanic forces declined with time of 50% ES as the MU fatigue index increased. (**F**) The ratio of the rheobase (Rh) current and input resistance plotted as a function of the after-hyperpolarization (AHP) half-decay time. The horizontal dashed line denotes the mean value of Rh/Rin of 3.0 in unoperated MG motoneurons. (**G**) Histograms of rheobase, AHP, input resistance and CV plotted as a percent of the total. Mean ± standard error (SE) values are shown in the plots in (**C**,**E**). Adapted from [31,32].

Increasing the daily activity to 50% of each day dramatically reduced the contractile force, speed and fatigability of the stimulated MG muscle (Figure 11A–C; [31]). The effects of the daily 50% were analogous to the fast-to-slow conversion of muscles and their fibers by continuous low-frequency ES regimens of Vrbová and Pette [22,23,24,25] and were accompanied by the almost complete fast-to-slow conversion of MusU types (Figure 13A; [31]). Chronic recordings of muscle contractions revealed rapid fast-to-slow muscle conversion, including the transition from “sag” to “non-sag’ of unfused contractions (Figure 14B,C; [31]). Acute intracellular stimulation and recording of MG motoneurons permitted a detailed study of their electrical properties in conjunction with the contractile properties of their MusUs, demonstrating a parallel conversion of motoneuron and MusU properties from fast to slow (Figure 14D–G). The distribution of MG nerve conduction velocities after daily 50% ES was shifted to lower values as compared with those of the nerves that were not stimulated, with their average values being significantly different (*p* < 0.05; Figure 14D; [31]). The time course of the slowing of conduction velocities during daily 50% ES (Figure 14E) paralleled the fast-to-slow conversion of their MusU properties (Figure 14C). More detailed study of the electrical properties of the motoneurons after 58–87 days of daily 50% ES confirmed their fast-to-slow conversion [32]. MG-type F and S motoneurons normally have Rh/Rin ratios of >7 and <7, respectively, with the ratio for the S-type drawn as a straight line in Figure 14F. The ratios for the stimulated motoneurons progressively declined to the S-type as the duration of the AHP increased to values typical of S-type motoneurons (Figure 14F; [32]). Indeed, the histograms of data collected for all the measures of motoneuron size, Rh, AHP, Rin and CV shifted to the values of S-type values (Figure 14G; [32]). Importantly, the size relationships between MU nerve and muscle properties remained after their F-to-S type conversion [31], with their demonstration being consistent with that in the cat soleus muscle in which all the motoneurons and their MusUs are slow [65].

#### 3.5.4. A Range of Properties Within MU Types

**Figure 15:**
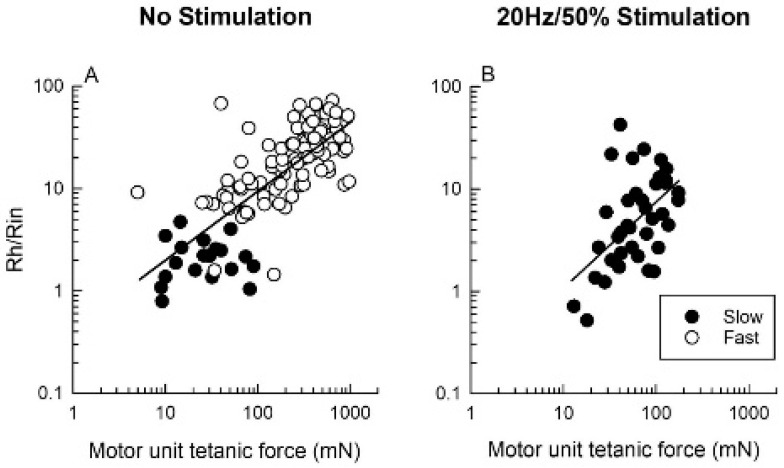
The correlation of motoneuron and muscle properties is retained after their fast-to-slow conversion. (**A**) The correlation of the ratio of rheobase (Rh) and input resistance (Rin) that differentiates slow from fast motoneurons with motor unit tetanic force is maintained after (**B**) 50% daily stimulation, where all motoneurons and their muscle units were classified as slow.

The experimental evidence of S-to-F conversion of nerve size when all neuromuscular activity was removed by SCI surgery [116] and the F-to-S conversion of muscle and their MusU properties by daily 50% ES [31,32], indicate the critical role of the daily *amount* of neuromuscular activity in determining the electrical properties of motoneurons, the size of their nerve fibers and matching properties of the motoneurons and their MusUs. The daily *amounts* of neuromuscular activities of <0.05%, 5% and 50% by ES converted the heterogenous MU populations in MG muscles to FF, FR and S types, irrespective of the frequency of the ES pulses (Figure 12, Figure 14 and Figure 15). Theoretically, if it were only the *amount* of neuromuscular activity that *determines* the properties of the motoneuron, motor nerve and muscle fibers that they supply, imposing any one of the controlled daily activities should result in restricted and narrow distributions of the properties of the motoneurons, nerves and MusUs. This was not the case, however, with the range of the motoneuron, nerve and MusU parameters remaining wide and frequently exceeding that of each of the MU types in the control unstimulated MUs. For example, despite the conversion of all the MUs to the S-type by daily 50% ES, the range of Rh/Rin values of MG motoneurons exceeded their normal range (Figure 14G and Figure 15; [32]). Moreover, the Rh/Rin ratios recorded in motoneurons after 50% ES increased as a function of the remaining but increased range of tetanic forces of their MusUs (Figure 15). Hence, the experimental findings indicate that neuromuscular activity has a strong *modulating* rather than a *determining* effect on the size and properties of motoneurons, nerves and MusUs. The expansion in the range of the electrophysiological measures of motoneuron and MusU size argue that other factors, in addition to the amount of daily activity, must be considered in determining MU properties.

Given the monosynaptic inputs on motoneurons that increase from S through to FF MUs [129], these inputs onto the motoneurons are likely important contributors to Henneman’s s1ize principle of the orderly recruitment of MUs according to size [63,64,67]. The fact that the size relationships between nerves and their MusUs return in partially denervated muscles [101] reinnervated muscles, even after cross-reinnervation [72,78,80,88,102,103,106,107] and in paralysed muscles and muscles subjected to controlled amounts of daily activity with ES [31,33,34,35,80], demonstrates the centrality of the size principle of neuromuscular organization. The properties of motoneurons, their nerves and MusUs may be modulated by their activity, including spinal cord transection, spinal cord isolation, limb immobilization and space flight [10].

#### 3.5.5. Plasticity of Human Muscle Fibers

Human skeletal muscles are heterogenous in their fiber type composition, as they are in animals [130]. They both exhibit plasticity, but unlike experiments in which a single pattern of amount of daily activity can be imposed on muscles to demonstrate plasticity in rats, rabbits and cats, it is not possible to control the *pattern* or the *amount* of daily activity in human muscles. Rather, exercise programs that include endurance and intermittent exercises have been used to investigate the plasticity of muscles of athletes, healthy subjects, elderly subjects and subjects with nerve and muscle injuries and disease, including diabetes and amyotrophic lateral sclerosis. The endurance exercises are characterized by repeated, sustained, low-intensity contractions for prolonged periods of time without fatigue [42,131]. Intermittent resistance exercises, in turn, involve low-frequency, high-intensity contractions against external resistance. The programs essentially utilize the lower and upper levels of orderly MU recruitment curve, with the endurance exercise activating the <30% of all MUs that are low-threshold fatigue-resistant MUs and the intermittent exercise programs activating the high-threshold fatigable MUs that constitute the upper 30% of the total numbers of MUs in most muscles (Figure 13B; [132]).

Human muscle fiber types are divided into slow-twitch type I and slow-twitch type II, as in animal muscles, both based on myosin ATPase histochemistry. The type II fibers in human muscles have been further differentiated into intermediate type IIa and type IIx, based on their oxidative-glycolytic and fast-glycolytic histochemical profiles [130]. The histochemical recognition of type I and II fibers has been the mainstay of many investigations of muscle plasticity in human subjects [133]. Of these, many investigators have used computer searches across numerous databases, including PubMed, SPORTDiscus, MEDLINE and Google Scholar, to determine the fiber type composition in muscles subjected to endurance and/or intermittent exercise programs. Thereby, the studies evaluate whether and how the intervention programs affect healthy individuals and those who suffer injury or disease. Nonetheless, other more substantive investigations have been performed with regard to the identification of myosin isoforms and determining blood flow within muscles and the location, density and function of intramuscular capillaries, numbers and function of mitochondria and the proliferation and behaviour of satellite cells in their studies on muscle plasticity [133].

The link between the muscle and muscle fiber properties with the patterns and/or amounts of neuromuscular activity was identified in studies of the muscle fiber types in athletes. The studies found that athletes whose muscles contain a preponderance of type I fibers are likely to enjoy success in endurance-type events such as road cycling and marathon running. Also, football players typically possess more type I fibers compared to rugby players and handball athletes, as do European football players whose muscles are comprised of greater proportions of type I fibers and who cover greater distances [134,135,136,137]. The proportions of type II fibers are higher than type I in athletes, who are more likely to succeed in track sprint cycling, power-type events (i.e., Olympic lifting) or cyclic movements that require high-frequency MU firing (i.e., track sprint running). The elite runners who compete and succeed in competitive sprinting events in competitions also tend to have greater proportions of type II muscle fibers.

These important observations on the proportions of muscle fiber types in athletes may be explained by experimental findings that have elucidated fiber-type conversions with concomitant changes in the muscle fiber properties. First, endurance exercises lead to type-II-to-type-I fiber-type conversion with increases in the slow myosin composition of the myofibrils [138], mitochondria [139], number and densities of capillaries in association with increased blood flow to the muscle fibers [140,141,142], the number of nuclei per muscle fiber and the satellite cellular pool [133]. Second, intermittent resistance exercise leads to muscle fiber hypertrophy of type II fibers when they are repeated weekly for 6 weeks [143] and both type I and II fiber hypertrophy when the length of time of the exercises is prolonged [144,145]. Hypertrophy has been attributed to the addition of sarcomeres and myofibrils in parallel [146], along with increased amounts of actin and myosin contractile proteins in the exercised muscles [147]. There is also a paradoxical increase in the number of myonuclei in the type II fibers that contributes to hypertrophy of the fibers by enhancing protein synthesis (see [133]).

Regarding the question of the *range of* muscle properties in athletes, as well as the range in muscle conversions in healthy young and elderly individuals and those affected by nerve injury or disease, by exercise programs, many of the studies report a range of properties using different outcome measures. As an example, there remained a range in sleep quality that was assessed by PSQI (Pittsburgh Sleep Quality Index) in older subjects who performed a variety of different types of exercise, including Pilates, yoga and Nordic walking [147].

## 4. Conclusions and Significance

Slow- and fast-twitch muscle fibers are localized to deep and superficial compartments in extensive studies of limb muscles in many animal species and in humans. Kernell, in particular, pointed out the functional significance of this localization. The slow-twitch muscle fibers that fire at continuous low frequencies, maintain low force levels for long periods of time, localize deep in muscles and surround the bones stabilize the limbs during standing. In the background of this stability, the progressive recruitment of the more forceful fast-contracting muscle fibers, which tend to fire intermittently at high frequencies, accounts for the progressively increased forceful contractions during movement. In addition to MU recruitment, modulating the MU firing rates controls the force production of the muscles.

The early and simple delineation of fast- and slow-twitch skeletal muscles, in association with findings of the intermittent high-frequency and continuous low-frequency firing characteristics of their motoneurons, was the basis for the extensive studies by Vrbová, Pette and colleagues to support the view that it is the *pattern* of activity that determines the properties of muscles and their muscle fibers. Henneman’s size principle, that the size and properties of motoneurons and their MUs are matched *and* that MUs are recruited in order of their size, was brought into perspective by their work on the functional role of the diversity of muscle properties and functions [148]. Kernell’s extensive recordings of muscle contractile and histochemical properties concluded that it is *not* the pattern of activity of MUs that determines their physiological, biochemical, immune and histochemical properties, but rather it is the daily *amount* of activity that determines their properties. Gordon’s work verified this view with the recording of motoneuron and MusU properties, in addition to physiological and anatomical verification. Nonetheless, findings that, despite the conversion of muscle and MU properties to S, FR or FF by 50%, 5% and 0% daily activity, the range of properties in each of these MU types is retained argues that factor(s) other than either, or both the *pattern* and *amount* of daily neuromuscular activity, must be considered as determining the properties of the motoneurons and their MusUs. Similarly, the range of the effects of endurance and intermittent exercise programs on healthy subjects and those suffering nerve injuries and disease is also consistent with the argument that factors other than the *pattern* or *amount* of neuromuscular activity must be determined.

## Abbreviations

MU	motor unit
S	slow
FR	fast-fatigue-resistant
FF	fast-fatigable
F	fast
SO	slow oxidative
FOG	fast-oxidative-glycolytic
FG	fast-glycolytic
MusU	muscle unit
FDL	flexor digitorum longus
SOL	soleus muscle
MG	medial gastrocnemius muscle
FDL	flexor digitorum longus muscle
TA	tibialis anterior muscle
ES	electrical stimulation
s	second
CP	common peroneal nerve
PerL	peroneus longus muscle
CT	twitch contraction time
FI	fatigue index
CV	nerve conduction velocity
TTP	time-to-peak force
Rh	Rheobase current
Rin	input resistance
AHP	after-hyperpolarization
EMG	electromyographic signal
T-f	relationship between T muscle tension and f the frequency of stimulation frequency
SCI	spinal cord isolation for 8 months
min	minutes
SE	standard error
